# The Reduction of Credit Risk in the Health Care Industry in China: Are We Returning to the Pre-COVID-19 Era?

**DOI:** 10.3389/fpubh.2021.835500

**Published:** 2022-01-20

**Authors:** Lei Yan, Sheng Tang, Haiyan Wang, Jianhao Gao

**Affiliations:** Business School, Zhejiang Wanli University, Ningbo, China

**Keywords:** COVID-19, modified KMV model, default distances, health care industry, GARCH model, threshold regression model

## Abstract

This study aims to evaluate the changes in the credit risk of the health care industry in China due to the COVID-19 epidemic by the modified KMV (named by Kealhofer, Mcquown, and Vasicek) model to calculate the default distances. We observe that the overall default distance mainly first decreased and then increased before and after the COVID-19 epidemic control in China; after the epidemic was controlled, the overall credit risk was reduced by 22.8%. Specifically, as shown in subdivided industries, health care equipment and health care facilities have larger credit risk fluctuations, while health care suppliers, health care distributors, and health care services have smaller fluctuations. These results can contribute to our understanding of why the COVID-19 epidemic in China could be controlled earlier, and software facilities are more important than hardware facilities in public health safety. Our methodological innovation is to use the GARCH (generalized autoregressive conditional heteroskedasticity) model and threshold regression model to modify the important parameters of the KMV model. This method has good accuracy in the Chinese environment.

## Introduction

The subject of the impact of COVID-19 (1–4) consists of a set of problems that are central to returning to the pre-COVID-19 era. Research on the reduction of credit risk in the health care industry under the market environment is helpful to judge from the stage of epidemic prevention and control to the stage of economic recovery in China. The epidemic prevention and control stage means a reduction in the number of people infected, accompanied by an economic recession due to the control implementation. However, in the economic recovery stage, there is an increase in output and a positive economic growth rate brought about by the market mechanism. There is a certain sequential relationship between the two stages, but the transition period may be very long or very short. The success of this transition has always lacked certain criteria for judging. It is also conducive to countries that have successfully controlled the epidemic to continue to provide help and advice to other countries (5). To judge economic recovery, a very important prerequisite is that the economic operation is under the market economy system. According to the China Health Statistics Yearbook, 80% of hospitals in China are public hospitals (6); these embody the nature of public welfare, undertake public services, and are non-profit-seeking health institutions (7). At the same time, because healthy behaviors decisions involve some choices with important health consequences such as diet, exercise, weight loss, which are widespread and closely connected with the market. They are an important barometer of market recovery. The manufacturers and suppliers of pharmaceuticals and medical devices are mainly market-oriented operations. We believe that the effectiveness test of government governance for epidemic control is not the best, and only market-level recovery is the real success of epidemic control. This background makes us pay more attention to the health care industry of listed companies whose main products are pharmaceuticals and medical devices with a high degree of marketization. Since the early 1980s, when the period of centralized economic planning ended and the country began to rely more on a decentralized market-based system, the Chinese health care system has undergone changes (8). The reform of market economy system in China is characterized by gradual progress, In 1984, the government promulgated the “Drug Administration Law.” In 2000, the Chinese government promulgated the “Opinions on Reforming Drug Price Management,” “Provisions on the Pilot Work of Centralized Bidding and Procurement of Drugs by Medical Institutions,” and “Medicine Bidding Agency Qualification and Supervision and Administration Measures.” These stipulate the entrance mechanism of the health care market. Since then, the health industry in China has gradually entered a period of rapid development, which has been in a relatively complete market state. And this background explanation is conducive to our explanation for the effectiveness test of epidemic control under market conditions. According to the State Statistics Bureau of China, there were 7,382 pharmaceutical industry manufacturing companies, with an annual operating income of 23,908 trillion yuan by December 2019. After the outbreak of COVID-19 in February 2020, China adopted a joint prevention and control strategy (9), and this policy led to short-term personnel flow control, blockages in commodity circulation and temporary suspension of workers (10). This circumstance is no exception for the health care industry's involvement in public health safety. Its supply chain credit risk will also be affected in most industries (11, 12). However, the development of the health care industry is closely related to public health security, involves people's well-being, and is related to the long-term stability of society (13). If a sudden epidemic causes credit risk, the impact will inevitably cause structural problems in the supply of medical equipment, protective tools, and special drugs. On the other hand, since the public health system has received great attention during the epidemic, this activity has enabled the health industry to recover more quickly than other industries (14). The combined effect of these two reasons is that the operating income growth of the health industry from February to July was negative. By the end of 2020, the operating income reached 24,857 trillion yuan, which achieved positive growth. This phenomenon can comprehensively reflect the particularity of the health industry by market mechanisms (11). This feature have existed in China before the outbreak of the epidemic, so that it is conducive to analyze the economic recovery under market system.

The particularity of the selection of the health care industry lie in the following reasons: from the perspective of profitability, the default risk of short-term loans is inversely related to the ability of a company to operate. In the COVID-19 epidemic, companies in the health care industry will be constrained by short-term funds and face certain credit risks. If they fail to repay short-term loans in a timely manner, they will face risks such as enforcement and bankruptcy. From a non-profit perspective, the health industry is closely related to public health safety. Society's attention to public health safety will help the health industry recover earlier and help to predict the impact faced by other industries. This issue needs to be understood from the marketization perspective of China's health care industry. The COVID-19 epidemic has not only affected the credit risk of the health care industry (11), but also reflected returning to the pre-COVID-19 era by the renormalization of credit risk in the health care industry. Since the epidemic was brought under control earlier, analyzing the impact of the epidemic on the health care industry will provide some reference for the development of the world health care industry. Why should we emphasize the prejudgment function of credit risk instead of market risk and structural risk? Compared with credit risk, market risk reflects losses caused by changes in market prices and volatility, such as equity risk, interest rate risk, currency risk, etc; the structural risk has certain national characteristics and is a manifestation of long-term stability in this society. Credit risk more reflects the identity of the borrower, which is an important factor in determining the value of credit. Although the risk is that of the lender and includes lost principal and interest, and disruption to cash flows, credit risk mainly arises when borrowers are unable or unwilling to pay. It can quickly and accurately reflect the short-term impact of the epidemic on borrowers. Combining the characteristics of the health care industry, using short-term defaults in credit risk as a sensitive variable for economic recovery can more timely reflect the impact of changes in the epidemic on the market economy. This is an important prejudgment for choosing credit risk to study whether the economy is recovering. In order to illustrate this relationship more clearly, suppose that credit risk is closely related to the resource X available to the borrower, and D is the repayable debt. When there is no epidemic, the amount the borrower repays to the lender is Y, then Y=min{X,D}, which can imply the increasing concave ordering. When there is an epidemic, the borrower gets the resource Xe. If X is better than Xe at random, Y is also better than Ye in the increasing concave ordering.

To reflect the trend of credit risk in the health industry before and after COVID-19, this article chooses the KMV model—a credit risk default prediction model named by Kealhofer, Mcquown, and Vasicek (15)—as the method of measuring credit risk. Compared with the Z Score (16) and O-Score (17), the KMV model provides more information than either of the two accounting-based measures (18). The reasons we choose the KMV model instead of other models are that the model uses data from the stock market and the calculation process uses the daily stock price volatility of different companies rather than historical data. This approach can be more dynamic, reflecting the current credit status of the company (19). However, when the model is applied in China, the mapping relationship between the calculated default distance and the expected default rate faces challenges. This difficulty is caused by the immaturity of the historical default database of Chinese companies, but the default distance can be used to directly measure the credit risk level, which can solve this problem. Meanwhile, due to the assumptions of the model and the actual situation of China's stock market, the traditional KMV model result might not be very accurate (20, 21), but we can modify it by improving the KMV model's reliability.

This study is designed to investigate the dynamic impact of COVID-19 on the health care industry and evaluates the trend of returning to the pre-COVID-19 era. The plan of this paper is as follows: First, we evaluate the impact of COVID-19 on the overall credit risk of listed companies in the health care industry in China. Next, based on the short-term impact of COVID-19 on the health care industry, we amend the KMV model for the short-term credit risk evaluation method. Then, we analyse the impact of credit risk in different subdivided industry in the health care industry, which is helpful to clarify the focus on rescue in the medical industry after the outbreak of the epidemic. In addition, based on the judgement of the credit risk of the health care industry in China under the market mechanism, this study explores whether the health care industry can be heading back to the pre-COVID-19 era. The highlights of our research are as follows: first, we distinguish the difference between epidemic control and economic recovery, and uses credit risk as a variable for economic recovery to a normal state. Second, based on the close connection with public health, as well as the universality of the market connection, the health care industry as the object of analysis can better illustrate the return of the economy to the market state. Third, our modified KMV model is more accurate and more suitable for China. Due to the incomplete historical default data of listed companies, the mapping relationship between the default distance and expected default rate is not clear, we use default distance is to quantify the level of credit risk. And due to the fluctuations of “spikes and thick tails” might not follow the lognormal distribution in the short term, we use the GARCH (generalized autoregressive conditional heteroskedasticity) model to correct the accuracy in the volatility. And for the default point easily affected by subjective factors, we adopt the volatility threshold regression model. Fourth, this article takes advantage of the trend in credit risk changes of the health care industry to explain why the short-term credit risk is high under the COVID-19 epidemic and why it has recovered quickly after the epidemic was controlled. Finally, the differences in the credit risk of the health care subdivided industry indicate the importance of soft power for short-term epidemic prevention and control, and the long-term need to increase the allocation of medical equipment.

The remaining parts of this paper are as follows: In section Literature Review, we review the current research literature in the field. In section Data Source and Methodology, we explain the empirical data sources. In section Empirical Results and Robustness Test, we propose in detail a modification method to the traditional KMV model. In section Conclusions and Discussion, we present the results of the research. Finally, the conclusions and discussion elaborate on the empirical results.

## Literature Review

Because public health is mainly concerned the health of whole populations rather than individuals (22), the market could be involved in the resource allocation of pharmaceuticals and medical devices. Although the market supply has “adjustment shortcomings” when responding to sudden external infectious diseases, the government supply is better than the market supply in timeliness (22). After the epidemic is over or in a routine state, it has a bias in favor of market solutions because we assume that consumers are fairly knowledgeable and can make the best judgements of their own interests. The aerosol generation process causes airborne droplets to be emitted from the patient's mouth to the surrounding environment, which is one of the main transmission routes of the COVID-19 virus (23). This outbreak struck with damage (24), including layoffs, salary cuts of up to 75%, and unpaid leave in a market mechanism (25). Correspondingly, this outcome will also cause violent fluctuations in the stock market (11). The global stock markets lost approximately USD 6 trillion within a week following the COVID-19 pandemic announcement (26). This research includes impacts on the entire economy (1, 24) as well as related industries; examples impacts are agriculture (3), corporate social responsibility (2), supply chain (11), and stock market reactions to related industries (14). On the other hand, advances in medical care have had a major impact on morbidity and mortality (22), and aerosol generation process isolation requires the use of a respirator mask, most commonly the FFP2 (N95), FFP3 or equivalent masks (27). After the epidemic is under control, the market mechanism will play a more important role in deciding resource allocation. A company's credit risk is a risk of default on debt that could result from a borrower failing to repay loans (28). A company's credit risk is not only related to its current financial status but also closely related to its future development trends (29). In an efficient market, higher levels of credit risk will be associated with higher borrowing costs (30). Thus, measures of borrowing costs can be used to infer credit risk levels by market participant assessments (31).

In terms of credit risk measurement, Bharath and Tyler (32) use the KMV model to explain the difference between the CDS (credit default swap) premium and the bond yield, proving that the KMV model has universal applicability in measuring the risk of a single enterprise. Tudela and Young (33) show that compared with the probit credit risk model, their results obtained by the KMV model are more accurate and forward-looking. Duffie et al. (34) showed that the out-of-sample prediction performance of the KMV model is better than that of other models. In the problem of modifying the KMV model, Zhang and Shi (35) used particle swarm optimization and fuzzy clustering to modify the KMV model, and the results showed that their FC-PSO-KMV (Fisher's criterion-particle swarm optimization-KMV) model provided more accurate predictions. Song et al. (36) found that the traditional KMV model cannot effectively distinguish between ordinary companies and ST companies and uses particle swarm optimization to adjust the KMV model.

In summary, despite the importance in the area, there has yet to be a study that examines the impacts of COVID-19 on the health care industry, and it would thus be of interest to evaluate the trend of returning to the pre-COVID-19 era. However, it is necessary to use a certain method to modify the model to improve its accuracy. The existing literature lacks at least the following research. First, it is difficult to solve the problem of mapping the default distance from the model output to the default probability. Mature markets have a large amount of historical default data to build a default database, but the historical credit default data of Chinese listed companies are not complete. Although it can be mapped with a normal distribution according to the initial assumption, this method will be severe in the short term due to having a small sample. The second is the correction of model parameters, especially the setting of default points. Different default points will lead to large differences in empirical results, and most samples have a short time span and numerous interference factors, which lack persuasiveness. Third, the volatility of stock prices often has a phenomenon of “spikes and thick tails,” which might not follow the lognormal distribution and does not conform to the assumptions of the traditional KMV model. To compensate for the above shortcomings, this paper uses the core parameter of the default distance to measure the size of the credit risk. We use the GARCH model and the threshold regression model to correct the equity value volatility and default point that need to be used in the KMV model to make it suitable for the national conditions of China. This approach is more compatible, which not only avoids the mapping problem caused by the immaturity of companies' historical default databases but also further improves the accuracy and reliability of the model results.

## Data Source and Methodology

### Data Source

This article uses the Wind database to select five subdivided industries of China's health care industry: health care distributors, health care services, health care facilities, health care equipment, and health care supplies. In order to ensure the scientificity of the empirical results, this study adopted a stratified random sampling method. That is to say, the survey objects are classified according to five subdivided industries, which not only guarantee the differences between each subdivided industry, but also ensure the consistency within each subdivided industry. In the specific operation, samples were drawn at a rate of 10% through the Excel random number function. Then, we eliminated companies that lacked relevant data, and finally retained 40 sample companies for research. In the first quarter of 2020, the health care industry in China was affected by the outbreak of the COVID-19 epidemic. On the one hand, some health care companies delayed the start of operations because transportation was blocked and personnel traffic was restricted. This circumstance has had a significant impact on the operation of the company. On the other hand, masks, safety clothing, and disinfectant companies are working overtime to face the shortage of protective materials caused by the epidemic. Since the second quarter, the domestic epidemic has been brought under control, and business operations have begun to be on track. From the fourth quarter of 2019 to the second quarter of 2020, during this period of time, companies' credit risk-related variables fluctuate significantly. Table 1 shows descriptive statistics of the sample. There is a significant difference in the volatility of the equity value, with the maximum volatility reaching 0.556 and the minimum being only 0.082. The debt value gap between the samples is large; this standard deviation of short-term liabilities in one period is 67.907, and long-term liabilities are only 9.245. The changes in short-term liabilities are much greater than those in long-term liabilities, which shows that the epidemic has a more obvious impact on enterprises over a short period of time. Although within three stages, the equity value of the sample is generally lower than the asset value, the degree of volatility of the equity value is significantly higher than the total asset value. This finding shows that the changes in the equity value during the epidemic are reflected in a timelier manner, which is also conducive in the short term. To explore the degree and trend of volatility, the sample time of this article is divided into three groups: the first group is the fourth quarter of 2019, the second group is the first quarter of 2020, and the third group is the second quarter of 2020, to correspond to the epidemic situation. The three stages are before, under, and after the outbreak of the epidemic.

**Table 1:** Sample related variables and descriptive statistics.

**Variable**	**Observation**	**Mean value**	**Standard deviation**	**Maximum**	**Minimum**
Equity value (in billion yuan)	120	13.110	15.389	65.066	1.296
Equity value volatility	120	0.226	0.096	0.556	0.082
Debt value (in billion yuan)	120	5.194	7.172	35.716	0.052
Short-term liabilities (in billion yuan)	120	4.517	6.791	34.050	0.049
Long-term liabilities (in billion yuan)	120	0.677	0.925	3.796	0.001
Asset value (in billion yuan)	120	18.272	17.806	73.633	1.539
Asset value volatility	120	0.171	0.103	0.530	0.025

This part mainly sets base parameters that must be used in the KMV model. First, we set the risk-free interest rate, which can be the 1-year treasury bond interest rate. The People's Bank of China band announces the interest rate of national debt several times within a year, takes the average interest rate from 2019 to 2020, and obtains r = 0.0249. Second, considering the time limit for debt repayment, because the main outbreak of the epidemic in China is at the beginning of 2020, this article selects the closing prices of each sample company's trading days in the fourth quarter of 2019, the first quarter of 2020, and the second quarter of 2020. The benchmark time points are as follows: December 31, 2019; March 31, 2020; and June 30. We use the time node of the first quarter as the company's debt repayment time limit, which is T = 0.25. Finally, the market value of the equity is defined as the total liabilities of the company. Because there are still limited shares in China, this article defines the equity value calculation method as the product of the sum of the number of tradable and non-tradable shares and the stock price, with the stock price determining the base date.

### KMV Model

This article revises the traditional KMV model for short-term credit risk. The inaccuracy of the company's market value fluctuation is changed based on the GARCH model. The threshold regression model is also used to dynamically and accurately measure the default point. The threshold variable is determined based on the long-term and short-term debt. The KMV model is an extension and application of the BSM (Black-Scholes-Merton) option pricing model. The company's creditor is equivalent to holding a risk-free bond with debt as the face value and short selling a company's assets as the underlying asset. The option amount is a put option at the exercise price, and the shareholder is equivalent to holding a call option with the company's assets. Assuming that the company's assets obey the Itô process (32, 37), the distribution law of assets can be obtained, followed by the company's expected default rate.

The operation of the KMV model can be divided into the calculation of the asset value and volatility, the determination of the default distance and the mapping of the expected default rate. If there is a loan quantity D, the payment time limit is T, the asset value is *V*_*A*_, and its volatility rate is σ_*A*_; then, the equity value *V*_*E*_ at the time of repayment is:


(1) $$\begin{array}{l} V_{E}\left(T\right)=\max\left[V_{A}\left(T\right)-D,0\right] \end{array}$$

Equation (1) can be regarded as the income of the European call option and the exercise price D and expiration T. The KMV model assumes that the asset value follows the standard geometric Brownian motion:


(2) $$\begin{array}{l} dV_{A}=rV_{A}dt+V_{A}\sigma_{A}dWt \end{array}$$

where *W*_*t*_ is the standard Brownian motion. The call option at a certain time t can be calculated by the BSM pricing formula:


(3) $$\begin{array}{l} V_{E}=V_{A}N\left(d_{1}\right)-De^{-r\left(T-t\right)}N\left(d_{2}\right) \end{array}$$

(4) $$\begin{array}{l} d_{1}=\frac{\ln\left(\frac{V_{A}}{D}\right)+\left(r+\frac{{\sigma_{A}}^{2}}{2}\right)\left(T-t\right)}{\sigma_{A}\sqrt{T-t}}\;and\\ \;d_{2}=d_{1}-\sigma_{A}\sqrt{T-t} \end{array}$$

In Equations (3) and (4), both *V*_*A*_ and σ_*A*_ are variables that cannot be directly observed in the market. To obtain these two parameters, they must be established with the known equity value *V*_*E*_ and equity value volatility σ_*E*_:


(5) $$\begin{array}{l} \sigma_{E}=\frac{N\left(d_{1}\right)V_{A}\sigma_{A}}{V_{E}} \end{array}$$

Thus, by combining Equations (3) and (5), the asset value and asset value volatility rate of each sample company can be obtained. The distance to default (DD) refers to the distance between the value of a company's assets and the critical value of a company's default. When the default distance is larger, the distance from the point of default is farther. The smaller the probability of default is, the lower the credit risk. When the value of the company's assets is less than the point of default, the default distance is negative, and it is considered that the company does not breach the contract (32, 33, 37). This approach is the analysis basis and conclusion support for the empirical results obtained later. The calculation method of the default distance of the KMV model is:


(6) $$\begin{array}{l} DD=\frac{V_{A}-DPT}{V_{A}\sigma_{A}} \end{array}$$

The point of default (DPT) in the formula is defined as the critical point, which refers to the critical situation of a listed corporation in default. A linear combination of short-term debt (SD) and long-term debt (LD) is used to determine the default point in the typical way:


(7) $$\begin{array}{l} DPT=SD+0.5LD \end{array}$$

Default behavior occurs when the value of the company's assets is less than the book value of the debt, according to the KMV model.

### Volatility Parameter Modification: Based on the GARCH Model

The standard KMV model's strategy for calculating equity value volatility is to estimate the next period based on the prior period's historical stock price. In truth, the stock market in China has “spikes and thick tails” with regard to price changes. Even if this pattern is not the case, they frequently fail to adhere to the KMV model's assumption of a normal distribution of stock prices, which is a historical estimating approach based on historical data. The precision of is not very high. As a result, this article modifies the KMV model by using the GARCH model to determine the volatility of the stock value. The expression of the GARCH (p,q) model is


(8) $$\begin{array}{l} \alpha_{t}=\sigma_{t}\varepsilon_{t} \end{array}$$

(9) $$\begin{array}{l} {\sigma_{t}}^{2}=\alpha_{0}+\sum_{i=1}^{p}\alpha_{i}{\alpha_{t-i}}^{2}+\sum_{j=1}^{q}\beta_{j}{\sigma_{t-j}}^{2} \end{array}$$

(10) $$\begin{array}{lll} \sum_{i=1}^{p}\alpha_{i} & + & \sum_{j=1}^{q}\beta_{j}\leq 1{,\alpha }_{0}>0,\alpha_{i}\geq 0,\beta_{j}\geq 0 \end{array}$$

where *a*_*t*_ is a set of time series, ε_*t*_ is an independent and identically distributed random sequence with a mean of 0 and a variance of 1, ${\sigma_{t}}^{2}$ is the conditional variance, α_0_ is a constant, and α_*i*_ and β_*j*_ are the parameters to be estimated. Equation (10) shows the constraints of the GARCH model, and they are also an important basis for judging whether the model is applicable.

Because the time series model is based on white noise that follows a normal distribution, the J-B test must first be used to judge the data's normal distribution. The ADF test must be used to rule out the possibility of data instability; third, the autocorrelation test must be used to test dependencies within the variables; and finally, the ARCH effect must be determined. We construct a GARCH (1,1) model of order 1 once the sample data passes all of the aforementioned tests:


(11) $$\begin{array}{l} {\sigma_{t}}^{2}=\alpha_{0}+\alpha_{1}{\varepsilon_{t-1}}^{2}+\beta {\sigma_{t-1}}^{2} \end{array}$$

### Default Point Parameter Modification: Based on the Threshold Regression Model

The default point is defined by the traditional KMV model as the sum of half of the company's short-term and long-term debt. An individual fixed-effect variable-intercept panel threshold model was presented by Hansen (38). Its main goal is to see if economic variables have structural changes within a particular range. To more accurately determine the point of default, we use a threshold regression model to alter the default point setting to make it more in line with the Chinese health care industry. As an example, consider the single threshold of two-mechanism panel data:


(12) $$\{\begin{array}{c} Y_{it}=\alpha_{i}+\beta_{1}X_{it}+\varepsilon_{it}\;q_{it}\leq \delta \\ Y_{it}=\alpha_{i}+\beta_{2}X_{it}+\varepsilon_{it}\;q_{it}>\delta \end{array}$$

where *q*_*it*_ is the threshold variable, δ is the threshold value, and the exogenous explanatory variables *X*_*it*_ and ε_*it*_ are independent and identically distributed. This article revises the linear relationship between the default point and the short-term and long-term liabilities in the KMV model and divides the default points into two groups for regression based on the threshold value. To prevent extreme values from affecting the data according to the nature of the default point, the assets of the sample companies are used as the explained variables of the model, and the long-term liabilities and short-term liabilities are used as the threshold variables, to construct the following equations:


(13) $$\{\begin{array}{c} DPT_{x}=\alpha_{1}+\beta_{1}SD_{x}+\beta_{2}LD_{x}+e_{x}\;\theta_{x}\leq \delta \\ DPT_{x}=\alpha_{2}+\gamma_{1}SD_{x}+\gamma_{2}LD_{x}+e_{x}\;\theta_{x}>\delta \end{array}$$

### Calculation Process

We take China National Accord Medicines Corporation Ltd. as an example to illustrate the calculation process of credit risk. From September 30, 2019, to December 31, 2019, the calculation of credit risk at this stage is witnessed. At this stage, the overall stock price fluctuates very little, except for individual points that fluctuate greatly. Autocorrelation and partial correlation tests must be performed once the sample has been reduced to a collection of stationary time series. At the 95 percent confidence level, the *P*-values that correspond to all observations' Q statistics are more than 0.05, which indicates that the time series does not exist. As a result of the significant correlation, the sample can be deemed to have passed the correlation test and to have met the conditions of the following phase in the GARCH model building. The calculation results of the GARCH model are


(14) $$\begin{array}{l} {\sigma_{t}}^{2}=0.000118+0.258967{\varepsilon_{t-1}}^{2}+0.427819{\sigma_{t-1}}^{2} \end{array}$$

where the coefficients and α_1_ and β are both >0, and α_1_ + β < 1, which meets the constraints of the GARCH model, and thus, it has a better fitting effect. According to the calculation formula of daily stock price volatility under the GARCH (1,1) model,


(15) $$\begin{array}{l} {\sigma_{n}}^{2}=\frac{\alpha_{0}}{1-\left(\alpha_{1}+\beta \right)} \end{array}$$

From the equation, the sample's equity value volatility rate is 0.1528 in the fourth quarter of 2019. The remaining sample operations are the same as those listed above. The values will be calculated using the old KMV model if the constraint requirements are not met or the test fails.

Next, we use threshold regression to calculate the default point of short-term liabilities and select short-term liabilities as the threshold variable. The *p*-value is 0.0300, which indicates that this indicator is set as the threshold variable, and the default point structure will change within a certain interval. The model will also have a better fitting effect. The test results of the threshold effect are shown in Table 2. The results of the threshold regression are shown in Table 3. When short-term debt exceeds the threshold, the default point's short-term debt coefficient is 1.286, and the long-term debt coefficient is 2.115; when short-term debt is less than the threshold, the default point's short-term debt coefficient is 3.150, and the long-term debt coefficient is 5.506.

**Table 2:** Threshold effect test of short-term liabilities.

**Threshold**	**RSS**	**MSE**	**F-stat**	**Prob**	**Crit10**	**Crit5**	**Crit1**
Single	1.68E+21	1.44E+19	32.68	0.03	26.4721	29.7322	38.3507

**Table 3:** Threshold regression test results.

**Threshold value**	• **4.9974**	
Observation interval	≤ 4.9974	>4.9974
Number of samples	85	35
Short-term debt factor	3.15	1.286
Long-term debt factor	5.506	2.115
Intercept	2.424	22.778

In summary, the default point after correction using the threshold regression model is


(16) $$\{\begin{array}{c} DPT_{x}=242369339.4+3.150SD_{x}+5.506LD_{x}\;SD\leq 499740000\\ DPT_{x}=2277760501+1.286SD_{x}+2.115LD_{x}\;SD>499740000 \end{array}$$

## Empirical Results and Robustness Test

### Empirical Results

The expected default rate is the result of comparing KMV with the historical default database based on the default distance. This paper selects the default distance of sample companies for analysis. Through the analysis of the trend, it can be found that after the outbreak of the epidemic, 33 of the 40 observed samples showed an upward trend in the default distance, of which 27 first decreased and then increased and 6 continued to decline. The average default distance of each subdivided industry has shown a clear trend of first declining and then increasing. Among them, the default distance of the health care equipment industry and health care facilities has changed significantly, while the credit of health care supplies, health care distributors, and health care services has fluctuated slightly. The health care supply industry has the least significant fluctuations and is less affected by the epidemic.

The expected default rate is the result of KMV's comparison with the historical default database based on the default distance. This paper selects the default distance of sample companies for analysis. Table 4 shows the changes in the average default distance of listed companies in the entire health care industry before and after the COVID-19 epidemic control in China. These companies were affected by COVID-19 in the first quarter of 2020, which shows that the default distance of these companies has decreased, and the credit risk has risen to varying degrees. In the second quarter of 2020, when the epidemic was initially under control, the credit risk of most companies had been reduced. Through the analysis of the trend, it can be found that 33 of the 40 observed samples showed an upward trend in the default distance, of which 27 first decreased and then increased and 6 continued to decline. The credit risk of the listed companies in the health care industry of China increased by 24.7% during the epidemic growth period. After the epidemic was controlled, the overall credit risk reduced to 22.8%. The default distance of these companies increased from 24.7% to a decrease of 22.8%, which is very close to returning to the pre-COVID-19 era. The health care industry has begun to become an economic competitive advantage. These results could be due to the following reasons: In the short term, it is affected by the company resumption time and employee arrival time, especially the arrival time of employees in major epidemic areas, and the production of upstream and downstream enterprises. Affected by factors such as recovery and sales promotion, the performance of health care companies was affected. When the pressure of capital turnover increased and the solvency of debt decreased, the credit risk increased. In the long run, starting from the second quarter of 2020, the domestic epidemic situation in China has stabilized, and production has gradually returned to normal. These companies could take overtime measures to make up for previous production losses. Society can begin to step up its investment in the health care industry, the public's health awareness increases, the demand for treatment increases, and the total demand for the health care industry increases significantly. Moreover, the general public also expects that the income of health care companies will effectively improve in the future, and the stock market will respond accordingly. At the same time, the financial situation of the enterprise improves, and the credit risk will also decrease.

**Table 4:** Default distance of health care companies.

**Field**	**Code**	**2019Q4**	**2020Q1**	**2020Q2**	**Trend**
Health care equipment	002082.SZ	3.275	1.411	2.157	Fall, rise
	002223.SZ	6.707	2.901	3.700	Fall, rise
	002432.SZ	2.418	1.405	2.093	Fall, rise
	002551.SZ	5.974	1.293	2.148	Fall, rise
	300562.SZ	1.943	2.141	3.088	Rise
	300633.SZ	4.035	3.162	3.670	Fall, rise
	300753.SZ	4.021	3.308	2.435	Fall
	600055.SH	5.814	2.234	2.754	Fall, rise
	600568.SH	1.285	0.464	3.797	Fall, rise
	600587.SH	1.135	1.253	1.418	Rise
Average		3.661	1.957	2.726	Fall, rise
Health care supplies	300677.SZ	0.166	1.863	2.384	Rise
	300791.SZ	3.547	3.292	1.513	Fall
	600529.SH	4.213	3.738	4.117	Fall, rise
	603301.SH	0.014	0.856	1.266	Rise
	603309.SH	2.976	2.343	1.526	Fall
	603880.SH	2.584	1.185	1.785	Fall, rise
	603976.SH	2.233	2.536	2.111	Rise, fall
	002382.SZ	3.639	1.990	2.630	Fall, rise
	002901.SZ	3.355	3.336	3.846	Fall, rise
	300003.SZ	4.602	3.460	5.443	Fall, rise
Average		2.733	2.460	2.662	Fall, rise
Health care distributors	000028.SZ	3.538	2.194	2.271	Fall, rise
	000078.SZ	8.316	2.576	0.367	Fall
	000411.SZ	0.799	5.512	9.675	Rise
	000705.SZ	8.820	4.403	7.817	Fall, rise
	000788.SZ	1.952	1.352	1.876	Fall, rise
	000950.SZ	1.626	0.821	1.151	Fall, rise
	000963.SZ	6.847	3.838	4.645	Fall, rise
	002462.SZ	0.025	2.662	2.830	Rise
	002589.SZ	1.073	4.432	0.649	Rise, fall
	002788.SZ	2.205	1.250	2.372	Fall, rise
Average		3.520	2.904	3.365	Fall, rise
Health care services	000150.SZ	1.738	1.396	0.963	Fall
	002044.SZ	4.432	4.221	3.582	Fall
	300244.SZ	3.014	2.998	3.618	Fall, rise
	603108.SH	2.392	1.552	1.818	Fall, rise
	603882.SH	3.975	2.957	3.601	Fall, rise
Average		3.110	2.625	2.716	Fall, rise
Health care facilities	000509.SZ	3.001	1.765	1.312	Fall
	000516.SZ	3.243	0.868	1.481	Fall, rise
	002172.SZ	1.639	3.583	7.935	Rise
	002173.SZ	2.797	1.160	1.778	Fall, rise
	600763.SH	4.696	4.197	6.563	Fall, rise
Average		3.107	2.061	3.027	Fall, rise

The average default distance of each subdivided industry has shown a clear trend of first declining and then increasing. From a structured perspective, although it is assumed that the uncertainty associated with default is entirely generated by the company's value. However, modeling defaults is equivalent to building a good model of the company's assets and determining when the latter will fall below existing liabilities. Under the influence of the epidemic, this default is unpredictable, but changes in the company's credit risk will be obtained soon after the epidemic is controlled. Due to the disclosure of the company's information, this will be a structural feature conducive to the credit risk of the subdivided industry. The specific conditions for each subdivided industry are as follows: In the field of health care equipment, the distance to default of 7 samples out of 10 samples decreased and then increased. The average distances to default in this field in the three stages were 3.661, 1.957, and 2.726. The average distance to default in the first to second stages decreased by 46.55% and increased by 39.29% in the second to third stages. In the field of health care supplies industry, the default distance of 5 samples out of 10 samples decreased first and then increased, and 2 samples kept decreasing. The average default distances in this field in the three phases were 2.733, 2.460, and 2.662. The default distance decreased by approximately 10% from the first to second phases, and the second to third phases increased by 8.21%. In the health care product distributor industry, the default distance of 6 samples out of 10 samples decreased first and then increased, and 1 sample continued to decrease. The average default distances in this field in the three stages were 3.520, 2.904, and 3.365. From first to second, the average default distance decreased by 17.5% during the period, and the second to third periods increased by 15.87%. In the health care services, the default distances of 3 out of 5 samples decreased first and then increased, while the other 2 samples continued to decrease. The average default distances in this field in the three stages were 3.110, 2.625, and 2.716. The average default distance decreased by 15.60% in the first to second stages and increased by 3.47% in the second to third stages. In the areas of the health care facilities industry, the default distance of 3 samples out of 5 samples decreased first and then increased, and 1 sample continued to decrease. The average default distances in this field in the three stages were 3.107, 2.061, and 3.027. In the first to second stages, the average default distance decreased by 33.67%, and the second to third phases increased by 46.87%. In summary, in the five areas of the health care industry, the range of default distances of health care equipment and health care facilities is relatively changed, which indicates that the epidemic has the greatest impact on the credit risk of these two areas. However, in the third stage, its credit risk was obviously improved. The health care supplies, health care distributors, and health care services have the least significant fluctuations, which indicates that credit risk in these areas is least affected by the epidemic and the degree of recovery of credit risk is also relatively small. The differences in the subdivided health care industry are mainly due to the following reasons. First, the joint prevention and control policies in China have enabled patients to realize the difference between elective surgery and necessary surgery, as well as the discrimination between primary care and essential care. The short-term impact of the COVID-19 epidemic is mainly manifested in the sharp reduction of elective surgery and primary care. On the one hand, the demand for health care equipment, such as ultrasonic diagnostic equipment, electronic endoscopy equipment, X-ray diagnostic equipment, dental diagnosis and treatment equipment, has decreased sharply. On the other hand, since most hospitals in the health care facilities industry are non-public hospitals, the number of patients in these hospitals has dropped significantly. These two significant characteristics have led to the relatively large impact of these two subdivided industries. However, after the epidemic was controlled, the needs of these two subdivided industries had to quickly return to their previous operating conditions. Their credit risk correspondingly returns to the pre-COVID-19 ear. Second, the control measures during the COVID-19 outbreak blocked the product supply chain and led to a significant decline in the performance of the health care industry distributors, which were dominated by pharmaceutical and pharmaceutical commercial circulation. This outcome also caused health consultations, health assessments, third-party medical inspections, and pathological diagnosis businesses in the health care service industry to lose a few businesses. After the epidemic was controlled, the demands of these two industries quickly returned to their previous operating conditions. Third, the epidemic has had few impacts on consumables, such as medical tape, medical gloves, and medical catheters, in the medical and health care supply industry because these health care companies are closely related to public health, such as medical catheters, respiratory equipment, and blood pressure monitoring, and have improved or maintained the performance of these companies. Finally, after the epidemic was controlled, the elective surgery and primary care market began to recover. The performance of these subdivided industries has gradually improved, approaching the level of normalization before the epidemic.

### Robustness Test

It is necessary to establish whether they are statistically significant to investigate the validity of the modified KMV model for credit risk measurement. The default distances of the sample enterprises are classified into three groups based on the current scenario in China. Group 1 was in the fourth quarter of 2019 (that is, before the outbreak), Group 2 was in the first quarter of 2020 (that is, when the epidemic broke out) and Group 3 was in the second quarter of 2020 (that is, after the epidemic was temporarily controlled). The Kruskal–Wallis test statistic after testing is 18.775, and the rank averages are 79.53, 47.45, and 54.53, which indicates that the second set of data is generally small, while the first set is generally larger; the two-sided test is progressively significant. Because the *p*-value is <0.05, the null hypothesis is rejected, which indicates that it is statistically significant. A pairwise comparison of the groups is required to determine whether the difference between the three sets is significant. The following are the comparison results in Table 5.

**Table 5:** Comparison of Groups.

**Two samples**	**Test statistics**	**Standard error**	**Standard statistics**	**Significance**	**Adj. significance**
Group 2-3	−7.705	7.778	−0.91	0.363	1
Group 2-1	32.075	7.778	4.124	0	0
Group 3-1	25	7.778	3.214	0.001	0.004

The first and second groups are the most significant, which indicates that at the start of COVID-19, the default distance of the sample companies was significantly shortened, and credit risk increased. The third group's default distance was greater than the second group, which indicates that epidemic control in China became more effective and that business operations gradually returned to normal. Factors such as the orderly resumption of work and production have reduced the company's credit risk. However, some organizations lack adequate anti-risk skills and must recover from the epidemic's effects, and the reduced effect is not readily apparent. The significance of the first and third groups of data is 0.001, which indicates a substantial difference between the two sets, and it also reflects that the impact of COVID-19 on health care companies is smaller than that of the second group.

It can be observed that the default distance measured by the modified KMV model is generally smaller than that of the traditional KMV model. The GARCH model optimizes the original stock price volatility algorithm, and the default point is established by short-term debt and long-term debt. Our modified KMV model makes default point setting more scientific and the measurement result more reliable by a threshold regression equation. It accounts for the sudden change in the default points as well as the distribution of short-term and long-term debt of the company.

## Conclusions and Discussion

Through the test of our modified KMV model and the validity test, this article summarizes the following research conclusions about the credit risk of China's health care companies under the COVID-19 pandemic. First, the effects of the COVID-19 outbreak in China are transient and limited. The credit risk of the health care industry has demonstrated a trend of first growing and then reducing before and after epidemic control. The transient deterioration and repair of credit risk in China's health care industry supports this conclusion. Second, the impact of COVID-19 on the credit risk of the health care industry has obvious industry characteristics. This phenomenon can be explained by the difference in the credit risk impact of different subdivided industries. Among them, the health care equipment and health care facilities industry are mainly affected by the sharp decline in elective surgery and primary care, and this impact is greater than that of the other three subdivided industries. However, the impact of health care supplies is not significant because their products are mostly medical consumables. Third, epidemic prevention and control is a systematic project. In the early stage of the epidemic, coordinated governance by the government is extremely important for epidemic prevention and control. To determine whether the epidemic is effectively controlled, the credit risk restoration of the health industry under a market mechanism has a certain degree of predictability. Fourth, to compare with the traditional KMV model, our modified KMV model for short-term credit risk assessment in China has two advantages in volatility rate and default point estimation. Fifth, special medical assistance programs for short-term cash flow shortages are necessary for the health care industry during the outbreak of the epidemic. The credit risks of most companies have increased to varying degrees during the epidemic, and they are relatively high. COVID-19 has a greater impact on a company's short-term debt. Generally speaking, the credit risk of health care industry comes from limited liability and resource constraints. Under the epidemic situation, this is more manifested as resource constraints, that is, enterprises face more constraints on the allocation of resource endowments.

The above research results provide inspiration. In the public health system, the basic principle of doing things is as follows: under the outbreak of the epidemic, we know that the government can solve the market supply “adjustment shortcomings” (20). The research in this article shows that after the epidemic is controlled, the characteristics of the market allocation of resources will be further developed. In other words, in the field of public health, after the epidemic, if the market and society can do well, the government should let the market and society do it; if the market and society cannot do things that no one wants to do, the government should do it. This is because soft power reflects the solution of short-term problems, which is suitable for the emergent characteristics of the epidemic. For public health, more health care equipment should be added to the prevention and control of the epidemic, and this must be resolved through the government. In addition, although the epidemic is uncontrollable, companies in the health care industry usually need to strengthen debt structure management in scale and maturity. These methods are the fundamental way to control corporate credit risk, although strict debt control and the scale of credit sales could affect the sales of products. At the same time, to prevent the short-term credit risk caused by the spread of the epidemic, the company should also strengthen the purchase of joint funds and insurance, which has resolved the deterioration of risks under uncertain conditions. These funds can consciously transfer potential losses and benefits to others with common economic interests. Certainly, the government should provide fund injection and financing for the health care industry because they involve public health safety.

## Data Availability Statement

Publicly available datasets were analyzed in this study. This data can be found here: https://www.wind.com.cn/NewSite/wft.html.

## Author Contributions

LY writing the paper and estimations. ST writing the paper and methodology. HW reviewing the paper and data collection. JG writing the paper and validation. All authors contributed to the article and approved the submitted version.

## Funding

This paper was supported by the Scientific Research and Innovation Team of Zhejiang Wanli University (Grant No. 202036) and the National Natural Social Science Foundation of China (Grant No. 21BGL228).

## Conflict of Interest

The authors declare that the research was conducted in the absence of any commercial or financial relationships that could be construed as a potential conflict of interest.

## Publisher's Note

All claims expressed in this article are solely those of the authors and do not necessarily represent those of their affiliated organizations, or those of the publisher, the editors and the reviewers. Any product that may be evaluated in this article, or claim that may be made by its manufacturer, is not guaranteed or endorsed by the publisher.
